# Overview of Systematic Reviews on Septic Arthritis of the Temporomandibular Joint (SATMJ)

**DOI:** 10.3390/jcm14030835

**Published:** 2025-01-27

**Authors:** Karolina Lubecka, Kacper Galant, Maciej Chęciński, Kamila Chęcińska, Filip Bliźniak, Agata Ciosek, Tomasz Gładysz, Katarzyna Cholewa-Kowalska, Dariusz Chlubek, Maciej Sikora

**Affiliations:** 1 Department of Oral Surgery, Preventive Medicine Center, Komorowskiego 12, 30-106 Kraków, Polandfblizniak@gmail.com (F.B.); 2 Faculty of Medicine, Medical University of Lodz, Al. Kościuszki 4, 90-419 Lodz, Poland; kacpergalant.ld@gmail.com (K.G.); agata.ciosek@stud.umed.lodz.pl (A.C.); 3 National Medical Institute of the Ministry of Interior and Administration, Wołoska 137, 02-507 Warsaw, Poland; maciej@checinscy.pl (M.C.); checinska@agh.edu.pl (K.C.); sikora-maciej@wp.pl (M.S.); 4 Department of Maxillofacial Surgery, Hospital of the Ministry of Interior, Wojska Polskiego 51, 25-375 Kielce, Poland; 5 Department of Oral Surgery, Medical College, Jagiellonian University, Montelupich 4, 31-155 Kraków, Poland; t.gladysz@uj.edu.pl; 6 Department of Glass Technology and Amorphous Coatings, Faculty of Materials Science and Ceramics, AGH University of Krakow, Mickiewicza 30, 30-059 Kraków, Poland; cholewa@agh.edu.pl; 7 Department of Biochemistry and Medical Chemistry, Pomeranian Medical University, Powstańców Wielkopolskich 72, 70-111 Szczecin, Poland

**Keywords:** septic arthritis, temporomandibular joint, temporomandibular joint disorders, systematic review

## Abstract

**Objectives**: This overview of systematic reviews was carried out following the PRIOR guidelines. It aimed to collect and compare the results of systematic reviews on the etiology, diagnosis, and treatment standards of septic arthritis of the temporomandibular joint. **Methods**: ACM, BASE, Google Scholar, PubMed, and Scopus were searched on 5 January 2025, for systematic reviews on SATMJ etiology and treatment. Records underwent selection, AMSTAR 2 evaluation, data extraction, and qualitative synthesis. **Results**: Three systematic reviews were included, covering 38 reports (93 cases), 37 reports (91 cases), and 25 reports (40 cases), respectively. There are seven source reports common to all three reviews. The reviews co-indicate possible odontogenic etiology, differ in opinions about the impact of chronic diseases, and agree on the superiority of pharmacotherapy, though without consensus on specific antibiotics. Severe complications of SATMJ, including potentially lethal ones, were reported. **Conclusions**: SATMJ is a serious condition requiring urgent and precise medical intervention, yet no clear management guidelines exist. The low overlap and inconsistency of the previous systematic reviews provide a foundation for a more comprehensive synthesis.

## 1. Introduction

Temporomandibular joints (TMJs) are responsible for the movements of the mandible [1]. They are located on both sides of the head; therefore, we distinguish the right and left TMJs. Both TMJs work together due to the continuity of the mandibular bone [1,2]. Thanks to their movement, it is possible to chew food and properly articulate sounds. These movements are complex during the act of chewing [3]. Abduction and adduction movements are projected onto the sagittal plane as a Posselt diagram [4,5]. As a Gothic arch diagram, the protrusion, retraction, and lateral movements are projected onto the transverse plane [4]. Each TMJ consists of a socket on the temporal bone and a head on the condylar process of the mandible. These surfaces are separated by the articular disc, a biconcave structure built of connective tissue. The articular disc divides the TMJ cavity into two compartments, upper and lower [1,6]. In the upper compartment, the sliding movement between the articular fossa and the anterior slope of the articular tubercle on the temporal bone, and the upper surface of the articular disc, predominates. In the lower compartment, the hinge movement between the lower surface of the articular disc and the articular surface of the head of the condylar process of the mandible is dominant. During mandibular abduction, the hinge movement precedes the sliding movement [7,8].

As a result of the coupling of both TMJs, the dysfunction of one of them disrupts the movements of the mandible in all directions [9,10,11]. TMJ dysfunctions are a very wide group of diseases. Among them, injuries, internal derangement, inflammation, and degeneration are distinguished [9,12]. Typically, TMJ inflammation is sterile and results from morphological changes such as disorders of the structure and function of the articular disc, changes in the composition of the synovial fluid, and overload of the cartilage of the disc and articular surfaces on the bones [12,13]. This results in increased levels of proinflammatory mediators, further deterioration of the lubricating properties of the synovial fluid, and, in a positive feedback mechanism, further degradation of the joint surfaces [14,15,16]. Chronic inflammation of the TMJ leads to degeneration of the joint surfaces [14].

However, this is not the only inflammatory mechanism affecting the TMJ. Septic arthritis of the TMJ (SATMJ) is observed much less frequently. It can technically be described as an empyema of the joint cavity or phlegmon that has spread to the TMJ cavity [17,18,19,20]. Typical etiological factors of SATMJ are *Staphylococci*, although numerous bacteria and even fungi have been identified in cultures from the content of the infected TMJ cavity [17,21]. SATMJ spreads most often from adjacent anatomical spaces, mainly from the oral cavity, but this is not the rule. The coexistence of diseases causing immune deficiency seems to be a significant risk factor, but their role requires detailed understanding [19,21].

Scientific knowledge about SATMJ is drawn primarily from case reports, with only a very limited number of systematic reviews summarizing them [18,20,21,22,23]. There are no clear guidelines for treatment, and the available protocols differ. Gathering all the knowledge on SATMJ is a significant challenge due to the need to develop guidelines for clinicians. It should be emphasized that SATMJ is so rare that most maxillofacial surgeons and otolaryngologists will encounter it episodically [24,25]. At the same time, it is worth remembering that this is an acute, potentially life-threatening, condition with a rapid course, which implies the need for immediate and appropriate treatment [21,26].

This overview of systematic reviews was conducted to evaluate the existing reviews on SATMJ, particularly with respect to their degree of overlap, risk of bias, and consistency of conclusions.

## 2. Methods

The planned systematic review was preregistered on 23 November 2024, in the PROSPERO International prospective register of systematic reviews (Centre for Reviews and Dissemination, University of York, York, UK) under number CRD42024613462. The PROSPERO submission contains the basic elements of the protocol presented in detail in this chapter.

The methods characterizing the overview of systematic reviews were based on the Preferred Reporting Items for Overviews of Reviews (PRIOR) guidelines and checklist [27]. The paper was registered in Open Science Framework under the identifier https://osf.io/ujky4 (accessed on 25 January 2025).

### 2.1. Eligibility Criteria

The overview included all identified systematic reviews that met the following PICOS criteria: (1) problem (P): addressed the issue of SATMJ; (2) intervention (I): included at least one primary study that discussed SATMJ treatment; (3) comparison (C): criterion waived; (4) outcomes (O): provided at least one type of conclusions; (5) settings (S): was a journal-published systematic review [28,29,30]. The detailed eligibility criteria are presented in Table 1. No time frame limit was applied.

### 2.2. Information Sources

To ensure the comprehensiveness of the planned systematic review, the following broad-scoped engines were used to search medical article databases: (1) Association for Computing Machinery, Guide to Computing Literature (ACM); (2) Bielefeld Academic Search Engine (BASE); (3) National Library of Medicine, National Center for Biotechnology Information, PubMed (PubMed); (4) Google Scholar; and (5) Elsevier Scopus (Scopus). The numbers of records available in each of the databases covered by the engines mentioned above are presented in Table A1. Preliminary searches were conducted in the second half of 2024, and final searches were completed on 5 January 2025.

### 2.3. Search Strategy

A detailed query was created based on preliminary searches using the terms “temporomandibular”, “septic”, and “arthritis”, along with the established eligibility criteria. The words “systematic” and “review” were added to restrict the results to the relevant article types. Finally, the following search strategy was applied: “(temporomandibular OR temporomandibularis OR TMJ) AND (joint OR articulatio) AND (arthritis OR inflammation OR inflammatory OR infection OR infections OR empyema OR abscess OR osteomyelitis) AND (septic OR suppurative OR pyogenic OR microbial OR bacterial OR gram-positive OR staphylococcal OR fungal OR viral) AND systematic AND review”.

### 2.4. Selection Process

The identified records were exported from the individual search engines as files containing bibliographic descriptions. These files were entered into the Rayyan automation tool (version 2025-01-05; Qatar Computing Research Institute; Doha, Qatar and Rayyan Systems; Cambridge; MA; USA). Deduplication was semi-automatically performed with the help of the aforementioned tool. In the first step, the software identified potential duplicates, and then the indicated records were manually checked and resolved (F.B.). In the next step, two of the authors (K.L. and F.B.) independently and blindly screened the records against the eligibility criteria, analyzing the content of titles and abstracts only. After completion of the work, the decisions were unblinded. In case of unanimous agreement or disagreement, the questionable record was promoted to full-text evaluation. In case of an uncontested rejection, which was the most common decision, the record did not advance to the next stage. Subsequently, full-text articles were retrieved. If the full text was not available, this fact was noted in the Section 3 of this paper. Full-text systematic reviews were then re-evaluated for compliance with the eligibility criteria (K.L. and F.B.), again independently and blindly. In case of disagreement, a third author (M.C.) assessed the disputed paper in the same way, and the final classification was decided by voting.

### 2.5. Data Collection Process

Two authors (K.L. and K.G.) independently extracted data into tables and spreadsheets. Google Workspace was used for this purpose (version 2024.06.28; Google LLC, Mountain View, CA, USA). The process was performed without the use of automation tools. In case of discrepancies at this stage, a third researcher (M.C.) had a casting vote.

### 2.6. Data Items

The following data were collected: (1) bibliographic data, (2) number of primary reports, (3) number of cases identified, (4) type of risk of bias assessment undertaken, (5) number of reports and cases excluded due to risk of bias, (6) variables subjected to quantitative analysis, (7) subgroups specified in the quantitative analysis, (8) conclusions from the systematic review along with a certainty rating, if available.

### 2.7. Risk of Bias Assessment

The risk of bias in systematic reviews was assessed using the AMSTAR 2 tool by a pair of authors (M.C. and K.C.), without automation tools [31]. In case of discrepancies in decisions, an independent assessment was made by a third author (F.B.), and the final decision was the result of a vote by all three authors. AMSTAR 2 evaluates 16 key domains related to the review’s methodology and reporting. We rated each domain as “Yes” or “No”, and an overall quality rating was assigned based on the cumulative findings. According to the AMSTAR 2 method, the weighting of individual domains for the final assessment was different [31]. The following issues are assessed:

1. Protocol registered before commencement: was the systematic review protocol registered prior to data collection to avoid reporting bias?
2. Adequacy of the literature search: was the literature search comprehensive, covering key databases and other relevant sources?
3. Justification for excluding individual studies: were the reasons for excluding studies stated and justified?
4. Risk of bias from individual studies: was the risk of bias for included studies assessed using a valid and standardized method?
5. Appropriateness of meta-analytical methods: if meta-analysis was conducted, were the methods appropriate and statistically valid?
6. Consideration of risk of bias in individual studies in results interpretation: did the authors account for study-level risk of bias when interpreting their findings?
7. Explanation for heterogeneity in results: were potential causes of heterogeneity explored if present in the results?
8. Discussion of publication bias: was publication bias assessed and discussed, including the use of tools like funnel plots?
9. Funding sources for included studies: were the funding sources of included studies examined to identify potential conflicts of interest?
10. Adequate description of included studies: were the characteristics of included studies described in sufficient detail?
11. Adequate description of excluded studies: were the characteristics or reasons for exclusion of studies clearly reported?
12. Robustness of statistical methods: were statistical analyses robust, and were sensitivity analyses performed?
13. Consideration of the population, interventions, comparators, and outcomes (PICOs): did the systematic review clearly define its PICO framework and evaluate relevant evidence?
14. Data extraction in duplicate: was data extraction conducted independently by at least two reviewers?
15. Conflicts of interest for the systematic review authors: were the conflicts of interest of the authors of the review declared?
16. Funding of the review: was the source of funding for the systematic review itself disclosed?

The final rating classifies the review as “High”, “Moderate”, “Low”, or “Critically Low”, reflecting the trustworthiness of its results.

### 2.8. Synthesis Methods

The synthesis was performed in tabular form for readability and ease of identifying gaps in the data. The conclusions from the individual systematic reviews were compared, taking into account the categories of (1) epidemiology, (2) etiology, (3) diagnostics, (4) treatment and follow-up, (5) prognosis, and (6) scientific recommendations. Google Workspace software was used (version 2024.06.28; Google LLC, Mountain View, CA, USA).

## 3. Results

### 3.1. Systematic Review Selection

**Figure 1:**
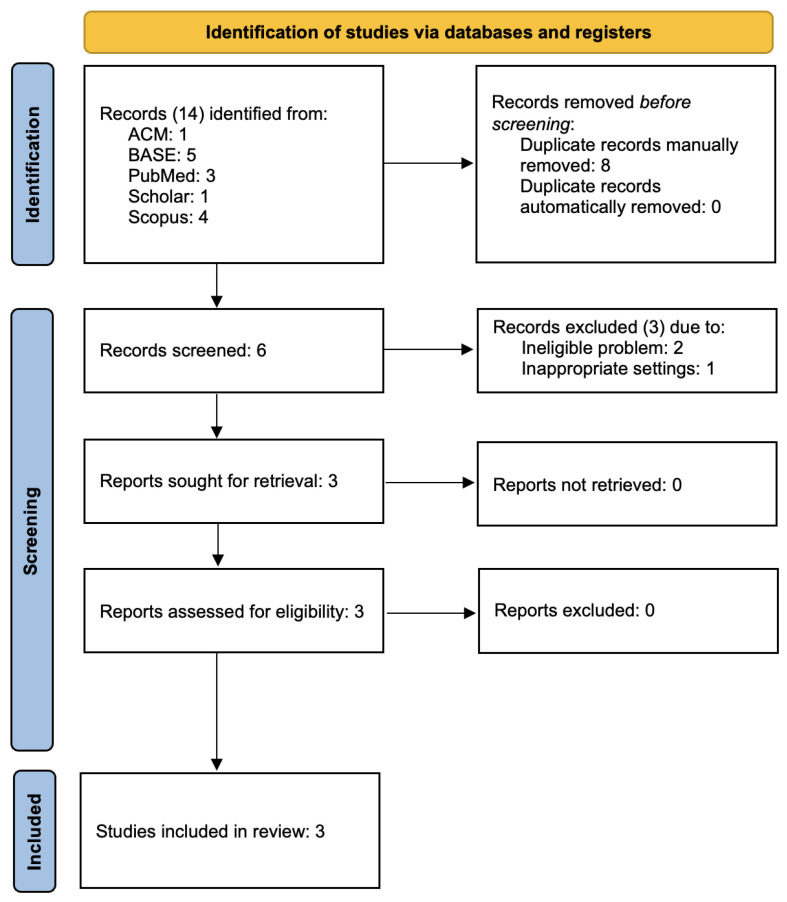
PRISMA 2020 flow chart.

The searches yielded 14 records in medical databases (one from ACM, five from BASE, three from PubMed, and four from Scopus) and one from a search of the gray literature using Google Scholar, with six remaining after deduplication. Two were unanimously rejected at screening due to an ineligible problem (Chappuis, 2023 and Röddiger, 2022), and one was rejected due to inappropriate settings (Luscan, 2016) [32,33,34]. The remaining three were retrieved in full text and eligible [21,22,23]. Figure 1 graphically presents the data review method.

### 3.2. Characteristics of Systematic Reviews

Three systematic reviews were incorporated into the overview (Table 2) [21,22,23]. These reviews include 38 reports (93 cases), 37 reports (91 cases), and 23 reports (40 cases), respectively. The papers are focused on collecting data on demographics, etiology, course, diagnosis, treatment, follow-up, and prognosis of SATMJ. In some source reports, in the course of the disease, inflammatory parameters, such as white blood cell count and C-reactive protein level, were assessed. None of the reports or cases included in the reviews were excluded due to a high risk of bias.

### 3.3. Primary Study Overlap

There are 7 source reports common to all three reviews, indicating a very low overlap of the primary studies (Table 3). The overlapping reports constitute 7 of 38 (18%) in the review by Omiunu et al., 7 of 37 (19%) in the synthesis by Jovanović et al., and 7 of 25 (28%) in the paper by Gera et al. [21,22,23]. The overlap between the two more extensively sourced systematic reviews (by Omiunu et al. and Jovanović et al.) consisted of 22 source articles (61%) [21,23].

### 3.4. Risk of Bias in Systematic Reviews

Based on the partial ratings for individual systematic reviews, the quality of each of the papers can be classified as “critically low” regarding the risk of bias. Each of them lacks at least one key domain (2, 4, 7, 9, 11, 13, or 15) in the AMSTAR 2 assessment (Table 4) [31]. Objectively good results in the remaining indices do not save the assessment, as they have a smaller impact on the quality evaluation. In particular, the deficiencies concerned domains 7 and 13, which refer, respectively, to the failure to provide reasons for rejecting primary studies and the failure to include an assessment of the risk of bias in primary studies when discussing the review results.

### 3.5. Synthesis of the Results

The conclusions from the systematic reviews are presented in Table 5. All of them suggest a possible odontogenic etiology. There is a contradiction regarding the significance of chronic diseases as a risk factor. Authors of the systematic reviews agree on the paucity of symptoms of SATMJ in the systemic dimension. There is agreement across all the reviews on the need to base treatment primarily on antibiotic therapy. However, the selection of a specific antibiotic remains an open question. The remaining conclusions were not common to all papers.

## 4. Discussion

Despite the existence of three systematic reviews on SATMJ, none of them is exhaustive, all have a high risk of bias, and the conclusions from them are moderately convergent [21,22,23].

The epidemiology of SATMJ is currently unknown, which could theoretically be changed by counting the annual number of reports of this disease [36,38,41,42]. This would provide information on the lower limit of incidence (the upper limit would remain unknown due to the presumption of unreported cases). In addition, a more meticulous assessment in this area would allow for the indication of continents and countries where SATMJ is reported more frequently. Such simple activities could lead to obtaining preliminary epidemiological data.

The etiology of SATMJ is almost exclusively bacterial, with the predominance of reports of the identification of *Staphylococcus aureus* in culture material [17,21,43]. Complications of inflammation originating from the oral cavity are mentioned first as the source of infection of the TMJ cavity [17,18,19,20]. However, this is not the only direction of the spread of the infection. There are known cases of otological complications that led to SATMJ [42,44]. There is a contradiction between the results of systematic reviews on the importance of systemic diseases as a risk factor for SATMJ.

The diagnostic process of SATMJ itself is not problematic. The local symptoms usually leave no doubt as to the identity of this disease [24,26]. Systemic manifestations of SATMJ are not common, and their occurrence may be associated with generalized infection or secondary involvement of the TMJ by infections primarily affecting other anatomical areas [20,24,42].

The included reviews consistently indicate systemic antibiotic therapy as the main therapeutic method for SATMJ [21,22,23]. Nevertheless, surgical treatment is considered a co-treatment, and its influence is currently difficult to assess. The importance of time is emphasized both in the context of the urgent implementation of treatment (primarily appropriate antibiotic therapy) and rehabilitation during the follow-up period after the end of treatment [21,22,23]. Due to the acute nature of SATMJ, the appropriate selection of empirical antibiotic therapy is important while waiting for the result of the microbiological test [21,26]. However, no single mandatory antibiotic has been indicated so far for administration as the first-line treatment [21,22,23]. In this context, vancomycin is being considered, which is related to the existence of cases of TMJ infection with methicillin-resistant *Streptococcus aureus* [24,43].

While SATMJ may have distant functional consequences for TMJ mobility, they are rare compared to the consequences of septic arthritis in other joints [17,26,42,45]. Chewing food alone is a rehabilitation procedure in the context of maintaining proper TMJ function during the period of convalescence after SATMJ [23,41]. Unlike fractures of the condylar process of the mandible, SATMJ does not require intermaxillary immobilization, which reduces the risk of future functional disorders [23,41].

The main limitation of this overview is its reliance on systematic reviews, which do not synthesize randomized controlled trials [21,22,23]. As a rule, guidelines for the management of medical sciences should be based on quantitative and qualitative syntheses of only randomized controlled trials [46]. In the case of rare diseases, this is more difficult, because the implementation of a randomized controlled trial is an organizational challenge. This would require a multicenter cooperation consisting of drawing the group to which the patient will be qualified and treating the established groups with different methods. In SATMJ research, such cooperation would have to be multi-year, because the expected incidence of this disease entity is approximately one case per several years per center [21,26,42,45]. To the knowledge of the authors of this paper, no such study has been conducted in SATMJ research.

What is available are primarily single-case reports and, occasionally, case series [18,24,26,36,38,41,42,43,44,45]. It should therefore be recognized that none of the qualified systematic reviews constitutes level 1 evidence according to the Oxford Center for Evidence-Based Medicine 2011 Levels of Evidence [47]. Despite this, the collected reviews bring us closer to obtaining holistic knowledge about SATMJ than papers on single cases [21,22,23]. The main advantages of the reviews summarized in this overview are their systematic nature, reliance on multiple cases, the assessment of the risk of bias, and the possibility of a full synthesis of the collected information. All of these reviews have shortcomings in the form of unsatisfactory coverage (as shown by the assessment of overlap of primary reports), lack of selection of source reports based on the conducted risk of bias assessment, and lack of detailed quantitative analyses [21,22,23].

Therefore, it is necessary to develop, conduct, and publish another systematic review of all known cases of SATMJ. The main assumptions of the new work would be its completeness in coverage of sources, applying of the risk of bias assessment in the selection of reports subjected to synthesis, conducting both qualitative and quantitative synthesis, and providing meta-analysis of numerical variables.

## 5. Conclusions

As a summary of the overview of systematic reviews, the following conclusions emerge: (1) the three identified systematic reviews, despite having similar publication dates, show limited overlap, leaving room for a more comprehensive systematic review; (2) the risk of bias, although assessed in the previous systematic reviews, was omitted in the qualification for the syntheses (this is the primary reason for the low-quality rating of the source systematic reviews); (3) the contradictions regarding the risk factors of SATMJ require scientific clarification; (4) the etiology and treatment of SATMJ were summarized in the previous systematic reviews without division into subgroups, which may be the reason for the discrepancies in conclusions drawn by the authors of the papers.

## Figures and Tables

**Table 1:** Eligibility criteria.

	Criteria for Inclusion	Criteria for Exclusion
Patients	Papers discussing SATMJ cases	Papers including animal studies or cadaver studies only
Intervention	Any conservative or surgical treatment presented	Treatment of only systemic diseases resulting in SATMJ provided
Comparison	Not required	Not applicable
Outcomes	Inclusion of conclusions from any of the following types: epidemiology, etiology, diagnostics, treatment and follow-up, prognosis, or scientific recommendations	Not applicable
Settings	Identification of the review as a systematic review in its title or abstract and inclusion of at least the eligibility criteria and search strategy in its full text	Preprints, non-journal publications

**Table 2:** Characteristics of included systematic reviews.

First Author, Publication Year	Number of Primary Studies	Number of Cases in Primary Studies	Risk of Bias Assessment Tool for Primary Studies	Reports/Cases Excluded Due to Risk of Bias	Quantitatively Analyzed Variables	Subgroups in Quantitative Analysis
Omiunu, 2021 [23]	38	93	Newcastle-Ottawa Scale	0/0	Age, sex, white blood cells, surgeries	None
Jovanović, 2022 [21]	37	91	Custom tool	0/0	Age, sex, hospitalizations, duration, mouth opening, white blood cells, C-reactive protein, pathogens, antibiotics, surgeries	None
Gera, 2023 [22]	25	40	Newcastle-Ottawa Scale	0/0	Age, sex, antibiotics, mouth opening, white blood cells, C-reactive protein, erythrocyte sedimentation rate, pathogen, surgeries	None

**Table 3:** Reports common to all three systematic reviews included in the overview.

First Author, Publication Year	Identifier
Araz Server, 2017 [35]	10.1016/j.ijom.2017.04.007
Ayachi, 2016 [36]	10.11604/pamj.2016.25.100.7943.
Cai, 2010 [37]	10.1016/j.tripleo.2009.08.007
Gams, 2016 [38]	10.1016/j.joms.2015.11.003
Lohiya, 2016 [39]	10.1016/j.joms.2015.06.166
Varghese, 2015 [40]	PMID: 25738723
Xiao, 2017 [41]	10.3892/etm.2017.4510

**Table 4:** AMSTAR 2 ratings [j].

First Author, Publication Year	1	2	3	4	5	6	7	8	9	10	11	12	13	14	15	16
Omiunu, 2021 [23]	✓	✗	✓	✓	✓	✓	✗	✓	✓	✗	✓	✗	✗	✓	✓	✗
Jovanović, 2022 [21]	✓	✓	✓	✓	✓	✓	✗	✓	✓	✗	✓	✗	✗	✓	✓	✓
Gera, 2023 [22]	✓	✓	✓	✓	✓	✓	✗	✓	✓	✗	✓	✗	✗	✓	✓	✗

✓—yes; ✗—no.

**Table 5:** Conclusions from previous systematic reviews.

Category	Omiunu, 2021 [23]	Jovanović, 2022 [21]	Gera, 2023 [22]
Epidemiology	SATMJ is a rare condition.	None	SATMJ is a rare condition.
Etiology	Contiguous spread resulting from odontogenic and otologic infections comprises a minority of SATMJ cases. Most patients do not have underlying conditions that affect immune function, such as diabetes mellitus.	SATMJ is a serious bacterial infection that occurs mostly in patients with chronic diseases. SATMJ often arises as a complication of dental infections or as a hematogenous metastasis from distant infectious foci. Gram-positive bacteria, particularly Staphylococcus aureus, are the most prevalent causative agents.	A small portion of SATMJ cases is caused by contiguous spread brought on by odontogenic and otologic diseases. The majority of people do not have underlying illnesses like diabetes mellitus that can impact immunological function.
Diagnostics	Systemic symptoms are uncommon, and most patients do not have leukocytosis.	Systemic signs of SATMJ are usually mild	Systemic symptoms are unusual. Most people do not have leukocytosis.
Treatment and follow-up	Broad-spectrum antibiotics are the cornerstone of treatment, often used in conjunction with arthrocentesis and other surgical interventions. Long-term follow-up care is crucial to prevent permanent joint dysfunction.	Causative bacteria are usually sensitive to vancomycin, which should be included in the initial antibiotic regimen to decrease therapy duration and avoid functional complications. Dentists should remain vigilant and ensure early administration of appropriate antibiotic therapy to prevent mismanagement.	The cornerstone of treatment is a broad-spectrum antibiotic, which is frequently combined with arthrocentesis and other surgical procedures. Long-term follow-up care is essential to avoid long-term joint dysfunction.
Prognosis	SATMJ may result in long-term adverse functional consequences. Unlike septic arthritis of other joints, SATMJ has a relatively low rate of adverse long-term functional sequelae with prompt and appropriate treatment. Mouth opening and jaw function improved in most patients after treatment.	None	SATMJ could have detrimental long-term functional effects. In contrast to septic arthritis of other joints, when promptly and appropriately treated, SATMJ has a relatively low rate of unfavorable long-term functional consequences. Most patients’ ability to open their mouths and move their jaws improves after treatment.
Scientific recommendations	Future studies with increased sample sizes are necessary to determine the efficacy of diagnostic modalities and ascertain the benefit of surgical interventions.	None	Further research with a larger sample size is required to assess the effectiveness of diagnostic tools and the value of surgical intervention.

## Data Availability

The original contributions presented in this study are included in the article. Further inquiries can be directed to the corresponding author.

## Appendix A

**Table A1:** Search engine scopes.

Engine	Scope, Records
ACM	3,815,653
BASE	411,948,582
PubMed	over 37,000,000
Scopus	over 94,000,000
